# Propolis

**Published:** 1867-10

**Authors:** 


### *Propolis.*

A private letter from Dr. Hitchcock, calls our attention to a typographical error in the preparation of this article, as set forth on page 419 of the JOURNAL. The proper proportion and method is as follows:—Propolis, ʒij. to ʒiv.; Liq. Potassæ, ʒj.

